# Preliminary observations of the urbanization and domiciliation of the American cutaneous leishmaniasis in Rio Branco, Acre, Western Amazon

**DOI:** 10.1590/0037-8682-0359-2022

**Published:** 2022-12-16

**Authors:** Andreia Fernandes Brilhante, Ricardo Andrade Zampieri, Eduardo Alcici de Souza, Ana Carolina Gomes Carneiro, Edmilson Pereira Barroso, Marcia Moreira de Ávila, Leonardo Augusto Kohara Melchior, Janis Lunier de Souza, Elizangela Soares de Oliveira, Maria Carmelinda Gonçalves Pinto, Lucile Maria Floeter-Winter, Eunice Aparecida Bianchi Galati

**Affiliations:** 1 Universidade Federal do Acre, Centro de Ciências da Saúde e do Desporto, Rio Branco, AC, Brasil.; 2 Universidade de São Paulo, Instituto de Biociências, São Paulo, SP, Brasil.; 3 Instituto Federal do Acre, Rio Branco, AC, Brasil.; 4 Universidade Federal do Acre, Centro de Ciências Biológicas e da Natureza, Rio Branco, AC, Brasil.; 5 Prefeitura Municipal de Rio Branco, Divisão de Entomologia e Bloqueio Químico, Rio Branco, AC, Brasil.; 6 Laboratório Central de Saúde Pública, Rio Branco, AC, Brasil.; 7 Secretaria de Estado de Saúde, Divisão de Vigilância Ambiental, Núcleo de Doenças Transmitidas por Vetores, Rio Branco, AC, Brasil.; 8 Universidade de São Paulo, Faculdade de Saúde Pública, Departamento de Epidemiologia, São Paulo, SP, Brasil.

**Keywords:** Health Surveillance, Leishmania, Psychodidae, Urban population

## Abstract

**Background:**

The American cutaneous leishmaniasis (ACL) is expanding in peri-urban environments.

**Methods:**

An entomological survey was conducted in the area of the occurrence of an autochthonous urban case of ACL. Sandflies and a parasitological slide of the human case were submitted for molecular diagnosis.

**Results:**

*Nyssomyia whitmani* and *Ny. antunesi* were the most frequently collected species. *Ny. whitmani* and *Bichromomyia flaviscutellata* were positive for *Leishmania guyanensis* and *L. lainsoni*, respectively. The human case tested positive for *L. lainsoni*.

**Conclusions:**

Sandflies and *Leishmania* parasites present in urban forest may occur frequently in nearby domiciliary environments; thus, these areas must be monitored.

In the state of Acre, American cutaneous leishmaniasis (ACL) is one of the most relevant diseases for public health, with cases reported in all municipalities, most of which are concentrated in the Vale do Acre region. Predominantly, the cases are of populations that live in rural and forest areas; when they live in urban areas, transmission occurs due to activities related to the forest^1^. 

Epidemiological studies in the region, involving entomological, human, and domestic animal surveys, detected the circulation of several *Leishmania* species in different hosts, and a high diversity of vector species, mainly in the rural and wild environments, was found^2^ ^,^ ^3^ ^,^ ^4^. Although, the presence of sandfly vectors in forest fragments in urban areas has been observed^5^; the circulation of *Leishmani*a spp. between vectors and human cases is linked to forest or rural areas. 

Changes in the environment and landscape may alter the dynamics of vectors and hosts and, consequently, the transmission of etiological agents. As ACL presents a high diversity of causative parasites and vector species, distinct epidemiological profiles may be identified in different regions of Brazil^6^ ^,^ ^7^.

In response to the demand from the Entomological Surveillance Service of the municipality of Rio Branco to clarify aspects of the transmission of an ACL autochthonous urban case in the Conjunto Universitário neighborhood, an entomological survey was carried out, aiming to identify the sandfly fauna and to investigate the presence of *Leishmania* DNA in female sandflies and in the human case. During three nights between August 27 and September 1, 2021, five Center for Disease Control and Prevention (CDC) automatic light traps were installed from 5:00 pm to 7:00 am in three environments: inside the house, in the kitchen, and in the hallway near the bedrooms (n=2); in the backyard, without the presence of domestic animals and vegetation, with the floor being completely cemented and clean (n=1), and in an urban forest fragment (approximately 100 m from the house) composed mainly of primary forest (n=2). Collection with a Shannon trap was undertaken in the backyard for one night, from 17:00 to 22:00 h, by two individuals.

The females were dissected for investigation of flagellated parasites in their guts, with subsequent molecular analysis. Undissected females and males were identified following the Galati taxonomic key^8^. 

The patient's clinical information is not reported here because it was not possible to access the data. According to information from the Entomological Surveillance of Rio Branco and the Central Laboratory of Public Health of Rio Branco (Laboratório Central de Saúde Pública de Rio Branco, LACEN-AC), the patient was a male, 21 years old, student, who had not visited forest areas in recent years. The patient presented with three lesions suggestive of cutaneous leishmaniasis on one of his legs, from which the amastigote forms were detected in the parasitological examination.

This project is part of the umbrella project “LeishAcre: Ecoepidemiological studies on leishmaniasis in Acre” and has been approved by the Ethics Committee in Research with Human Beings of the Federal University of Acre (CEP-UFAC) under the opinion number CAAE 26901619.4.0000.5010.

The Giemsa slide with a sample of the ulcer from the patient with a positive diagnosis and 41 dissected sandfly females were sent to the Institute of Biosciences of the University of São Paulo for molecular tests, which were performed using the high melting resolution (HRM) technique^9^. HRM approaches can detect differences in the melting profiles of PCR products as a result of variations in nucleotide composition, such as single nucleotide polymorphisms (SNPs) or other mutation types. The *hsp70* coding sequence is a polymorphic gene able to discriminate *Leishmania* species and, compared to most others, it is among the targets with the highest species-level discriminatory power^10^. Briefly, DNA samples were subjected to real-time PCR, in which three distinct hsp70 gene regions (amplicons 1, 2, and 3) were amplified independently. HRM analyses were performed at the end of each real-time PCR reaction. HRM profiles and specific melting temperatures (Tm) were determined and compared to the Tm obtained from DNA samples of reference strains of *Leishmania* spp.

During the entomological survey with CDC traps, 129 (58 females and 71 males) sandflies belonging to 14 species were collected (Table 1), and no insects were collected using Shannon traps. Of the 41 females submitted for molecular analysis, one of *Bichromomyia flaviscutellata* was positive, with a profile of *L. lainsoni* and three females of *Ny. whitmani* were positive, with a profile compatible with that of *L. guyanensis* (Figure 1). The positive specimens were collected in the forest fragment, and none of the 41 dissected females contained flagellated forms under bright field light microscopy. Regarding the slide of the human case, the HRM profile was compatible with *that of L. lainsoni* (Figure 1). 

**TABLE 1:** Sandflies in a residential area of Rio Branco municipality, Acre, Brazilian Western Amazon in 2021.

Species	Intradomicile	Peridomicile	Forest fragment	Total	%
*Bi. flaviscutellata*	-	-	2	2	1.6
*Br. avellari*	-	-	2	2	1.6
*Br. pentacantha*	-	-	2	2	1.6
*Ev. saulensis*	-	-	1	1	0.8
*Ev. walkeri*	1	-	5	6	4.6
*Lu. sherlocki*	-	-	4	4	3.1
*Ny. antunesi*	3	3	17	23	17.8
*Ny. whitmani*	-	6	58	64	49.6
*Pa. punctigeniculata*	-	-	1	1	0.8
*Pi. nevesi*	-	1	2	3	2.3
*Pi. serrana*	1	-	3	4	3.1
*Pr. calcarata*	-	1	5	6	4.6
*Pr. choti*	-	-	1	1	0.8
*Pressatia sp.*	-	-	9	9	6.9
*Vi. furcata*	-	-	1	1	0.8
**Total**	**5**	**11**	**113**	**129**	**100**

***Bi: Bichromomyia; Br: Brumptomyia; Ev: Evandromyia; Lu: Lutzomyia; Ny: Nyssomyia; Pa: Psathyromyia; Pi: Pintomyia; Pr: Pressatia; Vi: Viannamyia.***

**FIGURE 1:**
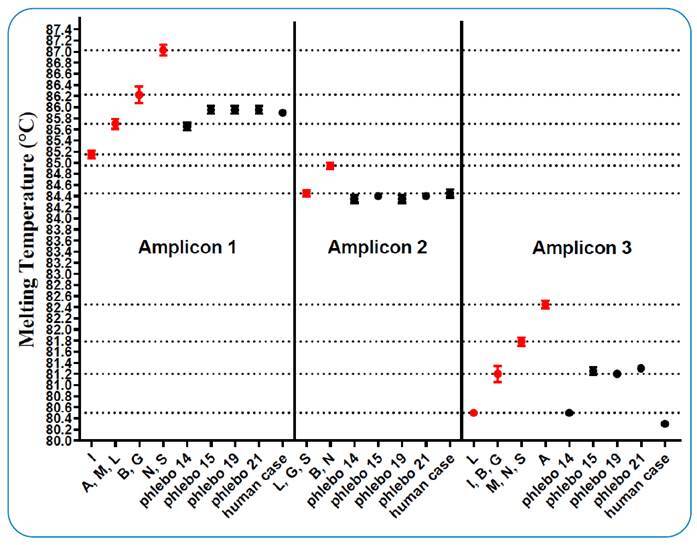
Melting temperatures obtained from HRM profiles. Average Tm values based on dissociation curves of *hsp70*-amplicons 1, 2, and 3 from the field samples (black dots) and *Leishmania* reference strains (red dots). Each species and sample were tested in duplicate. Reference strains: **I:** *L.* (*L.*) *infantum* (MCER/BR/1981/M6445); **A:** *L.* (*L.*) *amazonensis* (MHOM/BR/1973/M2269); **M:** *L.* (*L.*) *mexicana* (MNYC/BZ/62/M379); **L:** *L.* (*V.*) *lainsoni* (MHOM/BR/81/M6426); **B:** *L.* (*V.*) *braziliensis* (MHOM/BR/1975/M2903); **G:** *L.* (*V.*) *guyanensis* (MHOM/BR/1975/M4147); **N:** *L.* (*V.*) *naiffi* (MDAS/BR/1979/M5533); **S:** *L.* (*V.*) *shawi* (MCEB/BR/84/M8408). Field samples: phlebo **14:** *Bi. flaviscutellata*; phlebo **15, 19, and 21:** *Ny. whitmani*. Each species and samples were tested in duplicate and the data obtained were plotted using the GraphPad Prism v. 8.0.0 software.

In Brazil, ACL is expanding to peri-urban and urban environments, where human cases have been reported, and some vectors have been collected from households^6^. This scenario has also been reported in Argentina, a neighboring country^11^, where the adaptation of some sandfly species to urban and domestic environments has been observed owing to environmental changes and alterations. In this case, some species that were previously wild take on a peri-urban or urban profile under the pressure of these changes^12^.

In this study, the predominant species were *Ny. whitmani* and *Ny. antunesi*, the latter collected indoors, and the other species collected in the forest fragment near the residences. Since the first studies with sandflies in Rio Branco, species of epidemiological importance have been collected in urban environments and forest fragments, evidencing the urbanization process of some species such as *Ny. antunesi* and *Ny. whitmani* ^5^, which was found to be infected by parasites of the genus *Leishmania* in this study. These observations have also been reported in other cities in the Amazon region such as Belém^12^, Porto Velho^13^, and Sinop^14^.

For the detection of *Leishmania* DNA, most positive specimens were *Ny. whitmani,* emphasizing its high rate of infection. This species is recognized as a vector of ACL agents and is implicated in the transmission of three species of *Leishmania: L. braziliensis*, *L. guyanensis*, and *L. shawi*. In the last two species, *Ny. whitmani* acts as a maintenance vector for enzootic cycles^6^. This species is one of the vectors with the widest geographical distribution in Brazil and is considered a complex of cryptic species, which may have different behavior profiles in different Brazilian ecosystems, as well as a species that has been adapting and tolerating environmental changes^6^.

The presence of *Leishmania* DNA in *Bi. flavistutellata*, collected from a forest fragment, is reported here for the first time in Acre. In previous studies, this species has been reported in urban and peri-urban areas of Rio Branco^5^ and in forest areas in other locations of the state^4^. In Rondônia, a neighboring state *Bi. flaviscutellata* was found in greater density in preserved forests and with females engorged with human blood^15^, which demonstrates that this species is still dependent on the forest environment.

The human ACL in Acre has generally been attributed to rural and/or forestry cases, whether of individuals who live in these areas or those who occasionally visit these areas for leisure, ecotourism, or exploratory activities such as forest extraction and hunting^1^. It is also important to highlight that in the Acre state, six species of *Leishmania* cause dermotropic forms in humans, with the exception of *L. lindenbergi* ^2^. Thus, in this study, we reported the occurrence of the first autochthonous urban case of ACL in Acre, which was attributed to *L. lainsoni*, with the presence of incriminated sandflies species in the domestic environment and the circulation of vectors and *Leishmania* in the surroundings.

Thus, it is concluded that in Rio Branco City, some sandfly species frequent the domiciliary environment (indoor and peridomicile) in urban areas close to forest fragments; among them, *Leishmania spp.* vectors are suspected and recognized. Additionally, detection in forest fragments of sandfly females was positive for *Leishmania* spp*.* DNA indicates the need for periodic monitoring to verify the possibility of determining urban transmission foci. This preliminary observation serves as a warning for health surveillance in Rio Branco for decision making regarding preventive and control measures for this disease. Furthermore, the expansion of surveillance in other urban and peri-urban areas of the city is suggested.
